# Dissociative experiences of adolescents with borderline personality disorder: description and prediction

**DOI:** 10.1186/s40479-023-00217-0

**Published:** 2023-03-09

**Authors:** Mary C. Zanarini, Eduardo Martinho, Christina M. Temes, Isabel V. Glass, Blaise A. Aguirre, Marianne Goodman, Garrett M. Fitzmaurice

**Affiliations:** 1 McLean Hospital, McLean Hospital, 115 Mill Street, Belmont, MA 02478 USA; 2 Harvard Medical School, Boston, MA USA; 3 University of São Paulo, São Paulo, SP Brazil; 4 Massachusetts General Hospital, Boston, MA USA; 5 James J. Peters Veteran Affairs Medical Center, Bronx, USA; 6 Icahn School of Medicine, Mt. Sinai, New York, NY USA

**Keywords:** Borderline personality disorder, Adolescence, Dissociation

## Abstract

**Aims:**

The first purpose of this study was to assess the severity of dissociative experiences reported by adolescent inpatients with borderline personality disorder (BPD). The second purpose was to compare the severity of their dissociative symptoms to those reported by a sample of adult inpatients with BPD. The third purpose of this study was to assess a range of clinically meaningful predictors of the severity of dissociation in adolescents and adults with BPD.

**Methods:**

The Dissociative Experiences Scale (DES) was administered to a total of 89 hospitalized girls and boys aged 13–17 with BPD and 290 adult inpatients with BPD. Predictors of the severity of dissociation in adolescents and adults with BPD were assessed using the Revised Childhood Experiences Questionnaire (a semi-structured interview), the NEO, and the SCID I.

**Results:**

Borderline adolescents and adults had non-significant differences on their overall DES scores and subscale scores. They also had a non-significant distribution of low, moderate, and high scores. In terms of multivariate predictors, neither temperament nor childhood adversity was a significant predictor of the severity of dissociative symptoms in adolescents. However, co-occurring eating disorders were found in multivariate analyses to be the only bivariate predictor to significantly predict this outcome. In adults with BPD, however, both the severity of childhood sexual abuse and co-occurring PTSD were significantly related to the severity of dissociative symptoms in multivariate analyses.

**Conclusions:**

Taken together, the results of this study suggest that the severity of dissociation is not significantly different in adolescents and adults with BPD. However, the etiological factors differ substantially.

## Introduction

Dissociation has long been a clinical concern of those treating adults with borderline personality disorder (BPD) [1]. This concern is due to the suffering and functional impairment that clinicians often observe in their patients with BPD and dissociative symptoms. The prevalence of dissociative symptoms in adults with BPD has also long been a subject of interest to researchers. More specifically, 14 studies were published before 1990 that assessed the presence of the dissociative experiences reported by adults with BPD [2]. Taken together, it was found that these experiences were both common and discriminating for those with BPD when compared with adults with other psychiatric disorders.

More recently, a substantial number of studies have focused on the severity of the dissociative symptoms of adult borderline patients using the Dissociative Experiences Scale (DES) [3–5]—a 28-item self-report measure with proven psychometric properties. In general, they have found that borderline patients have a mean DES score in the moderate range (i.e., lower than the scores associated with trauma-related disorders) but higher than those in other diagnostic groups (e.g., schizotypal personality disorder, affective disorders, substance use disorders, and eating disorders).

It was also found that borderline patients had a significantly higher mean score on the DES than comparison subjects with other forms of personality disorders [6–8]. Additionally, it was found that borderline patients had higher mean scores than personality-disordered comparison subjects on the three factors that underlie the DES: absorption, depersonalization, and amnesia [6–8]. These three samples were also used to investigate the relationship between sexual adversity and the severity of dissociation among adults with BPD. One study found a strong relationship between this type of adversity and the severity of dissociation reported by a mixed sample of men and women with BPD [9]. The other two studies [10, 11] focused respectively on men and women with BPD and found no significant relationship between severity of dissociation and sexual adversity. Rather, in a sample comprised of those with BPD and those with another type of personality disorder, only the BPD diagnosis was a significant predictor of severity of dissociation.

In contrast, only three studies of the severity of dissociation in adolescents with BPD have been published [12–14] and all three used the adolescent version of the DES to assess the severity of dissociation [15]. Atlas & Wolfson [12] found that 26 female inpatient adolescents with BPD had significantly higher scores on the overall Adolescent DES mean score than 12 female inpatient adolescents with other psychiatric diagnoses (32.8 vs. 8.5). In addition, two other studies of mixed samples of outpatients and inpatients found significantly higher severity of dissociative symptomatology in adolescents with BPD compared to psychiatrically healthy adolescents [13, 14]. Koenig et al. [13] also found that their adolescent patients with BPD had a significantly higher mean DES score than psychiatrically ill comparison subjects.

Taken together, these earlier studies of adolescents only reported on the severity of the overall score of the DES, while the current study will also examine the severity of the three subtypes of dissociation and the three levels of dissociation assessed by the DES. This is important as these subtypes and levels of dissociation are qualitatively different from one another and have different treatment implications. In addition, these earlier studies were solely descriptive and did not include any predictor analyses of the severity of dissociation reported by adolescents with BPD. We have assessed predictors of severity of dissociation in both our adolescent and adult samples. We have studied childhood adversity factors, temperament, and co-occurring disorders that have their own clinical link to dissociation. This type of analysis is important to determine if the factors associated with the severity of dissociation reported by adolescents with BPD are the same as studies have found in adults with BPD.

As described below in the Methods section, our adult sample was collected 17 years before our adolescent sample. However, both samples consisted of inpatients who met the same rigorous, interview-based criteria for BPD, had the severity of their dissociation assessed by the same version of the DES, and had the same three measures administered to assess predictor variables. In addition, the difference in the years that each sample was collected provide a window into how changing parental and societal attitudes concerning the possibility of abuse inside and outside the family have changed over time.

The current study has three aims. The first is to describe the severity of the overall score of the DES, the levels of overall dissociation, and the severity of the three sub-scales of the DES reported by a well-defined sample of inpatient adolescents with BPD. The second is to compare their dissociative symptoms to those reported by a group of adult inpatients with BPD. The third is to study a range of clinically meaningful predictors of the severity of dissociation reported by adolescents with BPD. In addition, we will also study the same predictors of the severity of dissociation in our comparison sample of adults with BPD. We chose these three types of predictors of the severity of dissociation because either they were studied before in this context (childhood adversity), have been described as the core underlying aspect of BPD (Neuroticism), or have been recognized clinically as having their own dissociative features (co-occurring disorders).

## Method

The methodology of this study (the McLean/Mount Sinai Study of Childhood Development) (MSCAD) has been presented before in detail [16]. The institutional review boards at the participating institutions approved all study procedures. Briefly, all adults with BPD were inpatients at McLean Hospital in Belmont, Massachusetts who were admitted between June 1992–December 1995. Each patient was first screened to determine whether he/she 1) was between the ages of 18 and 35; 2) had a known or estimated IQ of 71 or higher; 3) and had no history or current symptoms of schizophrenia, schizoaffective disorder, bipolar I disorder, or an organic condition that could cause serious psychiatric symptoms.

Written informed consent was obtained from each patient. Then three diagnostic interviews were administered by research staff blind to their clinical diagnoses. The instruments were (1) the Structured Clinical Interview for DSM-III-R Axis I Disorders (SCID-I [17]), (2) the Revised Diagnostic Interview for Borderlines (DIB-R [18]; and (3) the Revised Diagnostic Interview for DSM-III-R Personality Disorders (DIPD-R [19]). The inter-rater and test-retest reliability of all three of these measures have been found to be good-excellent [20, 21].

Adolescents (aged 13–17) with presumptive BPD were recruited from four units at McLean Hospital and one unit at the Icahn School of Medicine at Mount Sinai between August 2007 and September 2012. For adolescent participants, parents provided consent and adolescents provided assent.

Adolescent participants, who all met the same inclusion/exclusion criteria concerning intelligence and co-occurring disorders as adult participants, were then administered the following diagnostic assessments during their index admission: (1) the Structured Clinical Interview for DSM-IV Childhood Diagnoses (KID-SCID [22]; (2) the DIB-R [18] and (3) the Childhood Interview for DSM-IV BPD (CI-BPD [23]). Inter-rater reliability of the KID-SCID and the CI-BPD have been found to be good–excellent [23, 24].

Dissociative experiences were assessed in both study groups using the Dissociative Experiences Scale (DES) [25]. The DES is a self-rating instrument comprised of 28 items that build on the assumption of a “dissociative continuum” ranging from mild normative to severe pathological dissociation, providing an overall score that has been normed in various clinical and non-clinical populations [26]. The scale also provides three sub-scores that have been found to be internally consistent: absorption, depersonalization, and amnesia [27]. Each item is rated according to the percentage of time that the patient has experienced that type of dissociative experience. For example, a score of 20 on the amnesia subscale of the DES suggests that the patient has spent 20% of his or her time having difficulties in remembering activities and relationships he or she might have had or in which he or she might have engaged. According to guidelines extrapolated from the developers of the DES [28], scores of less than 10 are considered to be in the low dissociation range, and scores between 10 and 29.9 are considered to be in the mid-range, similar to the range achieved by late adolescents and eating-disordered patients. Scores of 30 or above are in the high range, which is consistent with those of individuals meeting criteria for PTSD or dissociative identity disorder (DID).

Two other measures were also administered to both study groups. One was the Revised Childhood Experiences Questionnaire (CEQ-R) [29]—a semi-structured interview that assesses four types of childhood adversity: childhood sexual abuse, other forms of childhood abuse (verbal, emotional, and physical abuse), seven forms of childhood neglect (physical neglect, emotional withdrawal, inconsistent treatment, denial of subject’s feelings, lack of real relationship with parent or parental figure, caretaker placing subject in parental role, and caretaker’s failure to protect subject), and witness to violence (verbal, physical, and sexual). All four of these forms of adversity and their subcomponents were scored for severity. Sexual abuse scores ranged from 0 to 12. Other abuse scores ranged from 0 to 18. Childhood neglect scores ranged from 0 to 42. Witness to violence scores ranged from 0 to 9.

The other measure assessed temperament using Costa and McRae’s NEO Five Factor Inventory—a 60 item self-report measure that yields continuous scores on Neuroticism, Extraversion, Openness, Agreeableness, and Conscientiousness [30]. In these analyses, neuroticism scores were used as the core measure of temperament in those with BPD as it is linked to several emotions, such as anger and anxiety, that are significant in borderline symptomology [31].

### Statistical analyses

Between-group differences in demographic variables and predictor variables were assessed using Student’s t-test for continuous variables and Pearson’s chi-square for binary variables. Raw DES data was log transformed. Between-group comparisons concerning continuous dissociative symptomatology were conducted using linear regression and between-group comparisons concerning binary categories using logistic regression.

Analyses pertaining to comparisons of adolescents and adults with BPD were conducted controlling for sex and race. Linear regression analyses were used in the predictor analyses. All bivariate predictors with a p-level of < 0.05 were then analyzed in a multivariate model using backwards deletion to attain the most parsimonious model. The significance level for this model was set at < 0.01.

## Results

### Subjects

Two hundred and ninety subjects were adult inpatients at McLean Hospital who met both DIB-R and DSM-III-R criteria for BPD and all completed the DES. One hundred and four subjects were adolescent inpatients at McLean Hospital or Icahn School of Medicine at Mount Sinai who met both DIB-R and DSM-IV criteria for BPD and 89 of these subjects completed the DES. Due to staff error, the DES was not administered to 15 adolescents with BPD.

In terms of demographic characteristics (see Table 1), adolescents with BPD were significantly younger than adults with BPD. They were also significantly more likely to be female and non-white.

**Table 1:** Demographics of two study groups

	Adolescents BPD		Adult BPD		Adolescents BPD vs. Adults BPD	
	**%**	**N**	**%**	**N**	**χ**^**2**^	***p*-value**
Female	95.5	85	80.3	233	11.9	0.001
Nonwhite	33.3	17	12.8	37	5.0	0.025
	**Mean**	**SD**	**Mean**	**SD**	**t-test**	***p*-value**
Mean Age	15.7	1.3	26.9	5.8	−18.3	< 0.001

### Dissociative experiences

Table 2 describes the mean overall DES scores for adolescents and adults with BPD as well as the mean scores of the three factors derived from the DES. Adolescents and adults with BPD had non-significantly different overall DES scores and scores on its sub-components (absorption, depersonalization, and amnesia).

**Table 2:** Overall DES Score and DES Cluster Scores (mean ± SD) for two study groups

	Adolescent BPD (*N* = 89)		Adult BPD (*N* = 290)		Adolescent BPD vs. Adult BPD	
	Mean	SD	Mean	SD	*Regression Coefficient*	*p*-value
DES score	18.7	14.2	21.8	18.6	−0.14	0.963
*Absorption cluster*	27.1	19.2	29.1	19.2	−0.20	0.955
*Depersonalization cluster*	14.6	19.3	16.9	21.5	−4.51	0.208
*Amnesia cluster*	9.6	10.9	13.5	18.8	3.20	0.281

Linear regression, adjusted for sex and race, for comparisons between adolescents and adults with BPD

Adolescents with BPD, as indicated in Table 3, reported a substantial range of overall DES scores. About one third had a score less than 10, which is generally considered to be in the low range. About 45% had a score between 10 and 29.9, which has been found to be common in psychiatric diagnoses not primarily associated with trauma. The remaining one fifth had a score of 30 or higher, which has been found to be associated with PTSD and dissociative disorders, including DID. These results were not significantly different than those reported by adults with BPD.

**Table 3:** Severity of dissociative symptoms in adolescents and adults with BPD

Level of Dissociation Symptoms Severity	Adolescent BPD (*N* = 89)		Adult BPD (*N* = 290)		Adolescent BPD vs. Adult BPD	
	%	N	%	N	*Adjusted Odds ratio*	*p*-value
Low Levels (< 10)	34.8	(31)	31.7	(92)	1.05	0.883
Moderate Levels (10–29.9)	47.2	(42)	42.1	(122)	0.82	0.565
High Levels (≥30)	18.0	(16)	26.2	(76)	1.26	0.591

Logistic regression, adjusted for sex and race, for comparisons between adolescents and adults with BPD

### Predictor results

Table 4 describes and compares adolescents and adults with BPD on ten predictor variables. The four forms of childhood adversity all had significantly higher severity in the adult than the adolescent group. All five types of symptomatic disorders were significantly more common among adults than adolescents with BPD. Only neuroticism failed to distinguish between adults and adolescents with BPD.

**Table 4:** Descriptive statistics of predictor variables comparing adolescents and adults with BPD

	BPD Adolescents (*N* = 89)		BPD Adults (*N* = 290)		BPD Adolescents vs. BPD Adults	
	Mean	SD	Mean	SD	t-test	*p*-value
Childhood Adversity						
Severity of Sexual Abuse	0.29	0.59	1.79	2.18	6.38	< 0.001
Severity of Other Abuse	2.13	3.00	7.24	5.36	8.59	< 0.001
Severity of Neglect	6.00	6.75	14.6	10.67	7.17	< 0.001
Witness to Violence	1.72	2.02	3.42	2.50	5.87	< 0.001
Temperament						
Neuroticism	33.54	9.13	34.44	7.41	0.95	0.341
Axis I Disorders	%	N	%	N	χ^2^	*p*-value
Mood Disorders	91.0	81	96.9	281	5.51	0.019
Substance Use Disorders	38.2	34	62.1	180	15.78	< 0.001
PTSD	22.5	20	58.3	169	34.92	< 0.001
Anxiety Disorders	59.6	53	80.34	233	15.90	< 0.001
Eating Disorders	33.7	30	53.8	156	10.99	0.001

Table 5 focuses on the bivariate relationship between the ten predictor variables and the outcome of severity of dissociation in adolescents with BPD. The relationship between 10 predictor variables in three areas (childhood adversity, temperament, and co-occurring disorders) and the severity of overall dissociation reported by adolescents meeting criteria for BPD yielded four significant predicters at the *p* < 0.05 level: severity of childhood neglect, temperamental neuroticism score, history of a substance use disorder, and history of an eating disorder. However, the multivariate analyses resulted in only one variable with a p-level of < 0.01: co-occurring eating disorders (Values are the same as in Table 5).

**Table 5:** Bivariate predictors of severity of dissociation in adolescents with BPD

	Coefficient	*p*-value	95% CI
Childhood Adversity			
Severity of Sexual Abuse	0.13	0.967	(−6.24, 6.51)
Severity of Other Abuse	−0.004	0.994	(−1.01, 1.01)
Severity of Neglect	0.46	0.040*	(0.02, 0.90)
Witness to Violence	0.48	0.528	(−1.02, 1.97)
Temperament			
Neuroticism	0.40	0.016*	(0.07, 0.72)
Axis I Disorders			
Mood Disorders	2.56	0.634	(−8.00, 13.04)
Substance Use Disorders	7.03	0.022*	(1.02, 13.05)
PTSD	−2.00	0.581	(−9.21, 5.20)
Anxiety Disorders	5.61	0.068	(−0.41, 11.68)
Eating Disorders	9.79	0.002*	(3.77, 15.82)

Linear regression used in these analyses

Table 6 describes the same set of bivariate predictors and their relationship to the severity of dissociation reported by adults with BPD. It was found that the severity of all four forms of childhood adversity as well as co-occurring PTSD, anxiety, and eating disorders were all significantly related to the severity of dissociation reported by adults with BPD. The relationships between the Neuroticism score of the NEO and co-occurring mood and substance use disorders and the outcome of severity of dissociation in adults with BPD were not significant at the *p* < 0.05 level.

**Table 6:** Bivariate predictors of severity of dissociation in adults with BPD

	Coefficient	*p*-value	95% CI
Childhood Adversity			
Severity of Sexual Abuse	3.55	< 0.001*	(2.65, 4.45)
Severity of Other Abuse	0.85	< 0.001*	(0.46, 1.25)
Severity of Neglect	0.50	< 0.001*	(0.30, 0.69)
Witness to Violence	1.70	< 0.001*	(0.86, 2.55)
Temperament			
Neuroticism	0.05	0.712	(−0.24, 0.35)
Axis I Disorders			
Mood Disorders	3.89	0.538	(−8.55, 16.34)
Substance Use Disorders	1.39	0.539	(−3.06, 5.84)
PTSD	15.6	< 0.001*	(11.61, 19.59)
Anxiety Disorders	7.18	0.009*	(1.81, 12.55)
Eating Disorders	6.79	0.002*	(2.53, 11.04)

Linear regression used in these analyses

Table 7 describes the multivariate predictors of severity of dissociation in adults with BPD. Both the severity of childhood sexual abuse and co-occurring PTSD were significantly related to the severity of dissociation reported by adults with BPD.

**Table 7:** Multivariate predictors of severity of dissociation in adults with BPD

	Coefficient	*p*-value	95% CI
Childhood Adversity			
Severity of Sexual Abuse	2.39	< 0.001*	(1.38, 3.39)
Axis I Disorders			
PTSD	10.38	< 0.001*	(5.95, 14.81)

Linear regression used in these analyses

## Discussion

Our study has three main findings. First, borderline adolescents and adults were not significantly different in terms of mean overall DES score, mean DES factor scores, and in the distribution of overall severity scores. This is not surprising as they meet the same diagnostic criteria, of which dissociation is one. However, the fact that the severity of their dissociation is not significantly different lends even more evidence to the validity of the borderline diagnosis in adolescents as young as 13–17. Finding that there were no significant between-group differences in the overall score of the DES or its sub-scores gives added weight to the validity of the BPD diagnosis in adolescents as the BPD diagnosis has been found to be a valid psychiatric diagnosis in adults by the multifaceted criteria of Robins and Guze [32].

Second, facets of childhood adversity and the temperament aspect of neuroticism were not significant multivariate predictors of the severity of dissociation in adolescents with BPD. Only co-occurring eating disorders but not substance use disorders were significantly related to the severity of dissociation reported by adolescents with BPD.

While clinicians who specialize in the treatment of adults with BPD rarely link a co-occurring eating disorder to the severity of their patients’ dissociative symptoms, there are a number of research papers that do make this link [33–36]. More specifically, four research papers have found that binging is strongly associated with the severity of dissociation in eating disordered samples. The prevalence of binge eating disorder was substantial in both groups of patients with BPD—adolescents (21%) and adults with BPD (28%)—a non-significant difference [37].

Third, the severity of childhood sexual abuse seems to play a greater role in the severity of dissociation in adults with BPD than in adolescents with BPD. This may be due to the generally recognized fact that parents and society in general are far more hypervigilant about preventing the sexual abuse of children than they were a generation or two ago. Parents are not as trusting of extended family members, coaches, and neighbors as they once might have been and often limit unsupervised contact with these adults. In the same vein, children are taught to tell a teacher or school nurse if they are being abused at home or at school. In addition, teachers and school nurses are now mandated reporters of abuse. In addition, it may be that adolescents with BPD experienced forms of adversity that were less common when study adults were children or adolescents—being bullied or, particularly, being harassed on social media.

### Limitations

Limitations include that all subjects with BPD were initially inpatients. It may well be that borderline patients who have never been hospitalized have less severe dissociative experiences. It may also be that today’s adolescents have greater access to evidence-based treatments for BPD than adults who began their treatment histories before the development and/or dissemination of such treatments.

## Conclusions

Taken together, the results of this study suggest that the overall severity of dissociation, the severity of the sub-types of dissociation, and the prevalence of different levels of dissociation (low, moderate, and high) reported by adolescents with BPD are not significantly different than those reported by adults with BPD. In terms of predictive factors, the presence of an eating disorder centered around binging was significantly related to the severity of the dissociation reported by adolescents with BPD; a finding that is not surprising as reports of binge eating often include feelings of depersonalization and derealization. In contrast with adolescents with BPD, both the severity of childhood sexual abuse and co-occurring PTSD were significant predictors of the severity of overall dissociation reported by adults with BPD, who were initially assessed in an earlier time period. These differences in predictive factors may be due, at least in part, to differing levels of childhood adversity, which in turn, may be due to the more trusting vs. hypervigilant attitudes of parents and society in general in these different time periods.

## Data Availability

Requests for data must be made to Dr. Zanarini. Data is held in a secure cloud at Mass General Brigham, McLean’s parent company.
